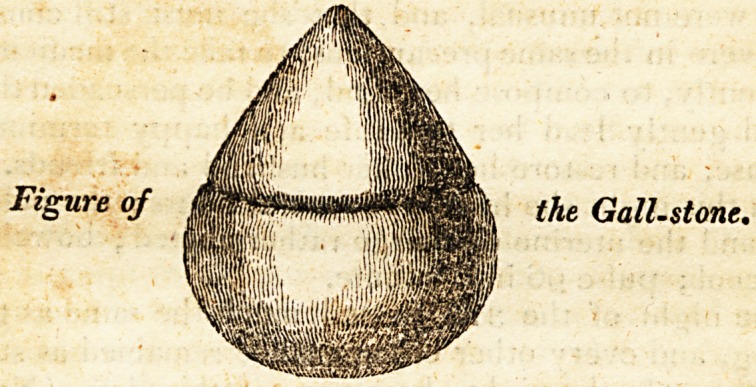# Symptoms of Apoplexy from a Large Gall-Stone

**Published:** 1818-06

**Authors:** Thomas Girdlestone


THE LONDON

### Medical and Physical Journal.

6 OF VOL. XXXIX.] JUNE, 1818. [NO. 232.

"For many fortunate discoveries in medicine, and for the detection of nume-
"rous errors, the world is indebted to the rapid circulation of Monthly
"Journals; and there never existed any work to which the Faculty in
"EUROPE and AMERICA were under deeper obligations than to the
"Medical and Physical Journal of London, now forming a long, but an
"invaluable, series."—RUSH.

*For the London Medical and Physical Journal.*

### *Symptoms of Apoplexy from a large Gall-stone;* by THOMAS GIRDLESTONE, M.D.

LAST summer, Mr. Clarke, a respectable farmer, near Halesworth, in Suffolk, came over to me, in consequence of his having experienced sudden sensations about his head, which alarmed his family, and left him with some degree of stupor, and great depression of spirits. He was naturally of a full habit, with a short neck, and had been subject to stomach complaints for more than a year before he was thus attacked. But, as he had a bloodless complexion, I suspected his disorder arose rather from a defective secretion of bile, than from any original disease in the head or stomach: and, as he was to return the next morning, I wrote a letter to Messrs. Walker and Howard, the medical gentlemen who attended the family of Mr. C., requesting them to examine the appearance of the motions, and recommending his perseverance in small doses of calomel, which I had ordered. The motions at first put on a clay colour: they improved in their appearance, under the medicine, and he ceased to experience any disagreeable sensation about the head: in every respect he thought himself much better, and persisted in taking the medicine, as originally directed, till after the middle of February, when he was seized with the most painful attack of gall-stones. Mr. Walker and Mr. Howard employed all the means they could think of to allay the sickness which the pain excited.—Glisters, with opium, were the only means by which the motions were produced, or any diminution of pain obtained. The motions continuing to be white, the jaundice increasing, and the patient deriving less and less relief from the injections,—I was desired to visit him on the 22d of February; and, about two hours before I reached the village, he experienced a sudden cessation from pain, followed by an incredible quantity of healthy-looking bilious motions, and the largest gall-stone I had ever seen pass by the intestines: it had a conical shape at one end, with a broad base at the other; and, not very distant from the middle of the stone, a circular depression appeared, as if the conical part had been for some time protruded, and the ring-like depression had been formed by the action of the duct on that part of the concretion; this part was much harder than the broader end, probably from its being beyond the reach of any bile, and it was about the colour of myrrh: the broader part was rather of a softer texture, and had more the colour of powdered rhubarb when moistened. I requested Mr. Walker to take it home, and give me its exact dimensions and weight.

He was very soon able to inform me of the perfect convalescency of our patient, and that the stone measured, round its shortest axis, three inches and a quarter; round its longest axis, rather more than three inches and three quarters,—and that its weight was one hundred and eighty-six grains. The gall-stone is in the possession of Mr. Clarke; but I can sketch it sufficiently accurate from memory, to give an idea of the ring-like contraction that was so evidently to be seen. Why the apoplectic symptoms should subside, on the motions resuming their natural colour, it is easy enough to understand; but it is not so easy, I apprehend, to explain why a gall-stone, of such a size and appearance, should have occasioned the decided pains of that disease only a few days before its expulsion. Larger biliary concretions than this have made their escape into the duodenum; but, I suspect, very rarely one of this size, in so short a space of time as six or seven days from the first unequivocal symptoms of the disease. The model of a gall-stone, mentioned by Dr. Pemberton, far exceeds this; but the patient suffered the most acute pains for five months, before it made its way into the duodenum. The extraordinary case, given by Mr. Thomas, in the sixth volume of the Medical and Chirurgical Transactions, was rather larger than that of my patient's, and weighed forty-two grains more. In Mr. Thomas's case, the rigors, local irritation, jaundice, and spasms, were absent; and this absence denotes rather a passive, than an active, state of the ductus cholidocus. But the symptoms which preceded the present described case of gall-stone, were those of violent pain, extending from the scrobiculus to the opposite part of the back, down to the hæmorrhoidal vessels, accompanied with vomiting and jaundice; all of which, with the form of the concretion, tend to prove that it was acted upon by the spasms of the duct.

In a patient, who died between seventy and eighty years of age, the thoracic and abdominal viscera were examined, at my request, in order to ascertain the cause of her death. Seventy-six gall-stones, of an uniform size, and dark green colour, were found loose in the gall-bladder, without the patient, during her long life, having ever experienced any symptoms of jaundice or pain about the biliary ducts. This case I had long since intended for your Journal, but I had laid it by so carefully, that I have only just succeeded in finding it. I shall therefore defer sending it to your Journal till next month.

*Yarmouth; May 5,* 1818.

*Figure of*  *the Gall-stone.*

---

*For the London Medical and Physical Journal.*

## Figures and Tables

**Figure f1:**